# Gene drives in our future: challenges of and opportunities for using a self-sustaining technology in pest and vector management

**DOI:** 10.1186/s12919-018-0110-4

**Published:** 2018-07-19

**Authors:** James P. Collins

**Affiliations:** Arizona State University, School of Life Sciences, Tempe, AZ 85287-4501 USA

**Keywords:** Gene drives, Values, Governance

## Abstract

Gene drives are systems of biased inheritance that enhance the likelihood a sequence of DNA passes between generations through sexual reproduction and potentially throughout a local population and ultimately all connected populations of a species. Gaps in our knowledge of gene drive systems prompted the US National Institutes of Health (NIH) and the Foundation for the NIH to ask the US National Academies of Sciences, Engineering, and Medicine (NASEM) to convene an expert panel to provide an independent, objective examination of what we know about gene drive systems. The report, “Gene drives on the horizon: Advancing science, navigating uncertainty, and aligning research with public values,” outlines our understanding of the science, ethics, public engagement, governance, and risk assessment pertaining to gene drive research.

Researchers have studied naturally occurring gene drive systems for more than a century. While CRISPR/Cas9 was not the first molecular tool considered to create an engineered gene drive, the advent of the CRISPR/Cas9 technology for gene editing gave a renewed impetus to developing gene drives in the laboratory for eventual release in the field. Recent experiments demonstrate that a CRISPR/Cas9-based gene drive can spread a targeted gene throughout nearly all of laboratory populations of yeast, fruit flies, or mosquitoes. Applying this basic science, there are proposals to use gene drive modified organisms to address such things as eradication of insect-borne infectious diseases and conservation of threatened and endangered species. Gene drives could potentially support agriculture by reversing pesticide and herbicide resistance in insects and weeds, and by control of damaging, invasive species. A major recommendation of the NASEM report is that there is insufficient evidence at this time to support release of gene-drive modified organisms into the environment. Importantly, the committee also recognized that the potential benefits of gene drives for basic and applied research are significant and justify proceeding with laboratory research and controlled field trials. This review summarizes highlights of the NASEM report with its focus on using the CRISPR/Cas9 genome-editing technology to develop gene drive modified organisms.

## Background

Advances in genetic technology continue to expand what is possible when it comes to altering the genes and ultimately the phenotypes of microbes, fungi, plants, and animals. These advances sharpen the debate around when and even whether we should develop and use new technologies to manipulate genomes. For example, imagine a proposal presented to a University Oversight Committee to develop a genome editing technology designed to reduce the size of mosquito populations that transmit pathogens that infect humans. Setting aside for a moment the reservations that some environmentalists and conservation biologists have when it comes to manipulating nature at all, the proposed research sounds like a potentially very useful application of biotechnology to reduce the burden of infectious disease in human populations. But what if the research proposal were to develop a genome editing technology designed to drive mosquito populations or even species extinct? The ethical implications of altering genomes for the purpose of extinction are greater than the implications of just population reduction. Both questions raise important issues regarding the ethical, legal, and social dimensions of this work, which sits squarely at the interface of science and society. Who governs altered organisms that can cross the boundaries of local, regional, and national jurisdictions? Where does regulation of such research start and stop? And who makes these decisions?

Gaps in our knowledge of gene drive systems prompted the US National Institutes of Health (NIH) and the Foundation for the NIH to ask the US National Academies of Sciences, Engineering, and Medicine (NASEM) to convene an expert panel to provide an independent, objective assessment of what we know about gene drives (Committee on Gene Drive Research in Non-Human Organisms: Recommendations for Responsible Conduct). The committee’s report, “Gene drives on the horizon: Advancing science, navigating uncertainty, and aligning research with public values,” summarizes our understanding of the science, ethics, public engagement, governance, and risks pertaining to gene drive research [1]. I address here three questions related to the report. 1) What are gene drives? 2) In what ways might values and governance change science and engineering methods, questions asked, and deployment of the technology related to using gene drives? 3) What are some ways forward given the importance of the ethical, legal, and social issues related to this research?

### What are gene drives?

Selective breeding is a traditional way to domesticate and then change further the traits of plants and animals. Efforts to manipulate the genes and chromosomes of organisms using various technologies extend from the early 1900s to present [2]. The process has generally involved imagining the possibility of changing genes, which dates back to de Vries’s mutation theory in the twentieth century’s first decade; harnessing a discovery as “a biotechnology” that could be applied, as for example in recombinant or genetically modified organisms; and then refining control of the technology.

Gene drives are systems that ensure biased inheritance by enhancing the likelihood a sequence of DNA passes between generations through sexual reproduction and potentially throughout an entire population [3]. Genes exhibiting what Burt and Trivers [3] called “drive,” occur in nature in many sexually reproducing species and are based on several kinds of molecular mechanisms [4]. By mid-twentieth century, there were proposals to harness and apply gene drives that went unfulfilled because the technology to alter genomes in ways that would result in spreading a trait throughout a population was unavailable. Austin Burt [5] gave a sense of the state of the field earlier in this century: “Site-specific selfish genes exploit host functions to copy themselves into a defined target DNA sequence….If such genes can be engineered to target new host sequences, then they can be used to manipulate natural populations....”

A global gene drive could enable a trait’s spread and persistence throughout a population or even an entire species assuming gene flow is sufficient. Sexually reproducing species with short generation times are most suitable for altering a population’s traits using a gene drive. Other characteristics include taxa in which resistance to the gene drive system is weak or does not arise at all [6, 7]. Finally, a species in which gene drive bearing organisms can disperse among most if not all sub-populations.

Many mechanisms expressed in a diversity of species are responsible for a gene exhibiting drive [3]. Champer et al. [4] compare and contrast the properties of five common gene drive systems: homing-based drives using homing endonuclease genes; sex-linked meiotic drives; Medea, the maternal effect dominant embryonic arrest system; underdominance or heterozygote inferiority drives; heritable microorganisms as illustrated by Wolbachia. CRISPR-Cas9 is a homing endonuclease based system that is the focus of the NASEM [1] report and of this review.

CRISPR-Cas9 is a technological advance in genome editing that provides a molecular tool for altering regions of DNA in ways that could yield a gene drive. As a gene editing method it is easier to use, faster to develop, and more precise than techniques such as zinc finger nucleases and TALENs [4, 8]. For a single locus homing endonuclease gene drive using CRISPR-Cas9 the drive cuts the wild-type chromosome. Ideally, the cell then copies the drive when it uses the drive-containing chromosome as a template for repair. All of an organism’s offspring repeat the process and inherit the drive-containing chromosome [9]. The speed and precision of genome modification have improved consistently over the more than 100 years of altering genomes using techniques such as x-rays, colchicine, radioisotopes along with other nuclear technologies, recombinant DNA, and now CRISPR-Cas9 [2].

The CRISPR-Cas9 technique offered a general method for spreading altered traits through non-human, sexually reproducing populations connected by gene flow [9]. This proposition put in sharp focus what was possible and added an urgency because of the relative ease and efficiency of employing the technology [10]. Engineered gene drives exemplify a technology with uncertain benefits and risks that raises compelling questions at the intersection of science and society.

### How will values and governance change science and engineering methods, questions asked, and deployment of the technology related to using gene drives?

Population suppression, resulting in a decrease in the number of individuals in a population, is one use of a gene drive system as is population replacement, resulting in a change in gene frequencies within a population [1]. Because global gene drives will spread throughout all populations of a species connected by gene flow and persist, gene drive-modified organisms hold the potential for yielding great benefits or harmful ecological change [10]. Researchers are working to develop and control CRISPR-based and non-CRISPR-based gene drive systems that are spatially or temporally limited in their spread to mitigate unintended consequences. For example, Buchman et al. [11] explore how reciprocal chromosome translocations might yield a high threshold gene drive through underdominance. This approach promises control via local, not global population replacement. Daisy-chain gene drives afford another means to alter just local populations, rather than inducing global change [12]. Daisyfield gene drive systems propose a temporal mechanism to limit spread [13], while Min et al. [14] propose a daisy quorum drive combining properties of the daisy chain model and underdominance. The aim here is limiting spread to a local area with the prospect of restoring the engineered population to its original genetic state if needed.

Several questions motivated the NASEM study including,

- Could global gene drives have unintended consequences for public health and the environment?
- Do we know enough to consider releasing gene-drive modified organisms into the environment?
- Should we use gene drive modified organisms to suppress or even eliminate pest species?
- How do we decide where to release gene-drive modified organisms?
- What role do governments have?

These questions fit within a general framework provided by the concept of anticipatory governance, which Guston [15] defines as a societal based capacity to manage emerging knowledge-based technologies while such management is still possible. The NASEM committee also called for a responsible science approach to developing and applying gene drive technologies, an approach calling for continuous evaluation, assessment, and education relative to social, environmental, regulatory, and ethical considerations surrounding gene drives. This argument also relates to responsible research and innovation, an emerging form of technology assessment dealing with technical aspects of biotechnology as well as societal and ethical issues [16]. Responsible science ultimately rests on values—deeply held, complicated, sometimes evolving beliefs about what kinds of things, in human lives and the world at large, should be fostered, protected, or avoided, and therefore about what people should and should not do [1].

While concluding that there was insufficient evidence to support the release of gene-drive modified organisms into the environment, the NASEM committee went on to recommend that laboratory research and controlled field trials should continue because the benefits of gene drives for basic and applied research are significant. Gene drives research, however, should follow a process that the committee imagined as having phases, each with risks and best practices [1]:

- Phase 0: Research preparation
- Phase 1: Laboratory-based research
- Phase 2: Field-based research
- Phase 3: Staged environmental release
- Phase 4: Post-release surveillance

Safety should be a major consideration at each phase with a special focus on two things [1]. First is confinement, which is using ecological conditions, such as climatic isolation, or biological methods to prevent unintended or uncontrolled persistence of an organism in the environment. Second is containment, which is using human-made or natural physical restrictions to prevent unintended or uncontrolled release of an organism (e.g., large cages, greenhouses, and aquaculture pens and/or geographic isolation). Beyond these considerations site selection criteria should include scientific and technical details such as presence of the target species; values of relevant publics; capabilities of local, regional, and national governance bodies; and ability of researchers to engage with local communities. All of this led to a recommendation to give preference to initiating tests in countries with the scientific capacity and governance frameworks to conduct and oversee the safe investigation and monitoring of gene drive-modified organisms, with a special focus on the capacity to assess the risk of developing and deploying such organisms [17].

Risk is the probability of an effect on a specific endpoint or set of endpoints due to a specific stressor or set of stressors [1]. In other words, how often would a specific change or changes in the environment affect something of value to society? Gene drive systems involve different molecular mechanisms and occur in a diversity of organisms, so it is reasonable to assume needing case-by-case studies of their effectiveness and analysis of risks. In particular, the committee recommended that researchers, regulators, and other decision-makers use the methods of ecological risk assessment to estimate the probability of immediate and long-term environmental and public health effects of gene-drive modified organisms. Ecological risk assessment is also a way to inform decisions about gene drive research, policies, and applications. Assessing risk should entail several steps: trace cause-and-effect pathways; identify sources of uncertainty; quantify the probability of the outcomes; incorporate concerns of relevant publics; compare benefits and harms; and compare alternative approaches [1]. Researchers, the NASEM committee argued, should also consider alternative methods to a gene drive system as the former might lack some of the negative elements associated with developing and deploying gene drive-modified organisms.

### What are some ways forward given the importance of the ethical, legal, and social issues related to this research?

The NASEM committee recommended that governing authorities, including research institutions, funders, and regulators, should develop and maintain clear policies and mechanisms for how public engagement will factor into research, ecological risk assessment, and public policy. But what participants—or publics—might require consultation as a condition for conducting gene drive-related research and eventual release of gene drive-modified organisms? How is their choice justified? How should we define such participants? The committee recommended three classes of participants: communities, groups of people who live in or near candidate release sites for gene drive organisms; stakeholders, people with direct professional or personal interests in gene drives; and publics, defined as groups of people who contribute to democratic decision-making but may lack direct connection to development and release of gene drive modified organisms.

Challenges related to control of gene drive research and development include the fact that existing governance mechanisms may be inadequate because they do not consider gene drives’ intentional spread and potential irreversible effects on ecosystems [18]. There may also be a lack of clarity in oversight jurisdiction, insufficient means for public engagement, or no policies for collaborating with other countries with divergent systems of governance. Regarding the latter, NASEM committee members concluded that the intellectual capital and research capacity of relevant institutions around the world should be expanded to facilitate appropriate knowledge exchange and research collaborations pertaining to development and deployment of gene drive-modified organisms. This includes building long-term relationships with scientists in low- and middle-income countries where field research on gene drive-modified organisms is likely to occur, especially in cases related to possibly mitigating the negative effects on human populations of vector born infectious diseases such as malaria or dengue [19].

Regulatory agencies with oversight authority over genome modification research should review risk assessment models and procedures to ensure that they capture the characteristics of organisms bearing gene drives. The NASEM committee recommended that the U.S. government clarify assignment of regulatory responsibilities for field releases of gene-drive modified organisms because of overlaps and gaps in U.S. regulation of organisms that are candidates for alteration with gene drives. After release, political boundaries do not limit spread of a gene drive-modified organism, but regulation of genetically modified organisms under the US Coordinated Framework for the Regulation of Biotechnology and United Nations Convention on Biological Diversity assumes containment. Research institutions, regulators, and funders should revisit international regulatory frameworks, national laws, non-government policy, and professional codes of conduct to determine whether to and how to apply them to gene drive-related research, development, and deployment. In general, greater attention needs to be devoted to the international implications of developing and deploying gene drive-modified organisms.

## Conclusion

The challenges for developing an innovation such as engineered gene drives are often seen as mainly or exclusively technical in nature; for example, What is the most effective and efficient way to modify an organism for the purpose of diminishing the size of or eliminating populations of human pests, such as mosquitos? However, developing such a technology cannot be separated cleanly from non-science and engineering-related issues such as who gets access to the technology, and why? Or even if the technology should be developed and deployed?

Decisions to deploy gene drive-modified organisms in the interests of human health, conservation, or increased agricultural productivity are invariably a product of local power relationships, cultural traditions, and social norms, among other factors. These issues and more will influence national and international governance of the development and deployment of gene drive-modified organisms.

There is also a view that we should not intervene in nature at all using a technology such as gene drive-modified organisms [20]. Reconciling this argument with proposals to use gene drives to relieve the burden of infectious disease in humans, conserve species, or increase agricultural productivity is not straightforward. Ultimately, reconciling such competing interests and values will determine how intrusive we are willing to be in shaping populations and ecosystems. It also highlights the importance of using a multidisciplinary or interdisciplinary approach for making decisions related to the development and application of gene drive technology.

The uncertain benefits and risks of gene drives calls for governance by a measured version of precaution that can be developed in a way that does not easily succumb to the common objections to it [21]. This paper and NASEM [1] make the case that precaution can be consistent with support for science. As we think about moving gene drive research into the future, the challenge is integrating the scientific freedom that allows research to move ahead with acting responsibly and conducting research that embraces ethical, legal, and larger societal values.

## References

[1] National Academies of Sciences, Engineering, and Medicine. Gene drives on the horizon: Advancing science, navigating uncertainty, and aligning research with public values. Washington, DC. The National Academies Press. 2016. doi: 10.17226/23405. 27536751

[2] Curry, H. A.. Evolution made to order. Plant breeding and technological innovation in twentieth-century America. University of Chicago Press, Chicago.2016.

[3] Burt A, Trivers R. Genes in conflict. The biology of selfish genetic elements. Cambridge: Harvard University Press; 2006.

[4] Champer J, Buchman A, Akbari OS (2016). Cheating evolution: engineering gene drives to manipulate the fate of wild populations. Nat Rev Genet.

[5] Burt A (2003). Site-specific selfish genes as tools for the control and genetic engineering of natural populations. Proc Biol Sci.

[6] Champer J. R., R. Reeves, S. Y. Oh, C. Liu C, J. Liu J, A. G. Clark, and P. W. Messer. Novel CRISPR/Cas9 gene drive constructs reveal insights into mechanisms of resistance allele formation and drive efficiency in genetically diverse populations. PLoS Genet 2017; 13(7):e1006796. 10.1371/journal.pgen.1006796 PMC5518997 28727785

[7] Bull JJ, Malik HS (2017). The gene drive bubble: new realities. PLoS Genet.

[8] Gaj T, Gersbach CA, Barbas CF (2013). ZFN, TALEN, and CRISPR/Cas-based methods for genome engineering. Trends Biotechnol.

[9] Esvelt, K. M., A. L. Smidler, F. Catteruccia, and G. M. Church. Concerning RNA-guided gene drives for the alteration of wild populations. eLife. 2014. 10.7554/eLife.03401. 10.7554/eLife.03401 PMC4117217 25035423

[10] Oye KA, Esvelt K, Appleton E, Catteruccia F, Church G, Kuiken T, Lightfoot SB-Y, McNamara J, Smidler A, Collins JP (2014). Regulating gene drives. Science.

[11] Buchman AB, Ivy T, Marshall JM, Akbari O, Hay BA. Engineered reciprocal chromosome translocations drive high threshold, reversible population replacement in drosophila. BioRχiv. 2016; 10.1101/088393. 10.1021/acssynbio.7b00451 29608276

[12] Noble C, Min J, Olejarz J, Buchthal J, Chavez A, Smidler A, DeBenedictis E, Church G, Nowak M, Esvelt K. Daisy-chain gene drives for the alteration of local populations. BioRχiv. 2016; 10.1101/057307. 10.1073/pnas.1716358116 PMC6486765 30940750

[13] Min J, Noble C, Najjar D, Esvelt K. Daisyfield gene drive systems harness repeated genomic elements as a generational clock to limit spread. BioRχiv. 2017; 10.1101/104877.

[14] Min J, Noble C, Najjar D, Esvelt K. Daisy quorum drives for the genetic restoration of wild populations. BioRχiv. 2017; 10.1101/115618.

[15] Guston D (2014). Understanding ‘anticipatory governance. Soc Stud Sci.

[16] Gregorowius D, Deplazes-Zemp A (2016). Societal impact of synthetic biology: responsible research and innovation (RRI). Essays Biochem.

[17] Brown DM, Alphey LS, McKemey A, Beech C, James AA. Criteria for identifying and evaluating candidate sites for open-field trials of genetically-engineered mosquitoes. Vector Borne Zoonotic Dis. 2014;14:291–9. 10.1089/vbz.2013.1364 PMC3993056 24689963

[18] Charo RA, Greeley HT (2015). CRISPR critters and CRISPR cracks. Am J Bioeth.

[19] Lavery JV, Harrington LC, Scott TW. Ethical, social, and cultural, considerations for site selection for research with genetically modified mosquitoes. The American Journal of Tropical Medicine and Hygiene. 2008;79:312–8. 18784220

[20] Global Justice Ecology Project staff. 30 environmental leaders say “no” to gene drives in conservation. 2016. http://globaljusticeecology.org/?s=gene+drives [accessed May 18, 2018].

[21] Kaebnick GE, Heitman E, Collins JP, Delborne JA, Landis WG, Sawyer K, Taneyhill LA, Winickoff DE (2016). Precaution and governance of emerging technologies. Science.

